# Effectiveness of Gentamicin Wound Irrigation in Preventing Surgical Site Infection During Lumbar Spine Surgery: A Retrospective Study at a Rural Teaching Hospital in India

**DOI:** 10.7759/cureus.46094

**Published:** 2023-09-27

**Authors:** Ayush Agrawal, Manoj K Ramachandraiah, Arun H Shanthappa, Sandesh Agarawal

**Affiliations:** 1 Orthopaedics, Sri Devaraj Urs Medical College, Sri Devaraj Urs Academy of Higher Education and Research, Kolar, IND

**Keywords:** effectiveness, wound irrigation, surgical site infection, lumbar surgery, gentamycin

## Abstract

Background: Surgical site infections (SSIs) are an opposing result of surgery and account for the majority of healthcare-related infections worldwide. It is one of the most common complications associated with open-spine surgery and is associated with high rates of mortality and high demand for healthcare resources. Surgical site infections are the result of a variety of reasons, which is why a range of prevention strategies have been proposed. Intraoperative wound irrigation (IOWI) is a simple procedure that involves moving a solution through an open wound to help hydrate the tissue. It is a type of prophylactic wound irrigation. It removes and dilutes bodily fluids, bacteria, and cellular debris. It may also act as a bactericidal agent when used with antibiotics and antiseptics.

Aims and objectives: To evaluate the incidence of SSI in lumbar spine surgeries by comparing IOWI with normal saline containing gentamicin (NS-G) and normal saline (NS) alone.

Materials and method: A hospital-based retrospective study was conducted among 40 patients who underwent elective lumbar spine surgery at the Department of Orthopaedics, RL Jalappa Hospital Centre, Kolar, Karnataka, India.

Result: Out of the total participants enrolled, 60% were males and 40% were females. There was no statistically significant difference found between mean age, mean BMI, mean hemoglobin level, mean WBC counts, and mean fasting blood sugar (FBS) levels among both groups. The overall prevalence of SSI among patients was 25%. In Group A (NS-G), the prevalence of SSI was 15%, and in Group B (NS), it was 35%. In total, 17.5% of study participants had superficial SSI, while 7.5% had deep SSI.

Conclusion: Gentamicin, an aminoglycoside antibiotic, is bactericidal and efficient against gram-positive organisms like *Staphylococcus*, the most frequent pathogen causing SSI in spine surgery. During lumbar spine surgery, IOWI with saline and gentamicin before closure is more effective in preventing SSI than simple saline irrigation.

## Introduction

Surgical site infection (SSI) is a common postoperative complication that causes significant morbidity, hospitalization, and mortality [1]. The global pooled SSI rate was 2.5%, with a 95% confidence interval (CI) of 1.6 to 3.7. The subgroup analysis based on WHO regions and survey period had an SSI rate of 2.7%, with a CI of 2.2% to 3.3% and 2.5% to 3.5%, respectively [2]. In low- and middle-income countries, SSI is the most frequent type of hospital-acquired infection and affects up to one-third of the patients who undergo any surgical procedure [3].

Likewise, SSI has been identified as one of the most frequent complications of open spine surgery, resulting in considerable morbidity, mortality, and burden on healthcare resources [4-6]. Surgical site infection rates for spinal surgical procedures range from 0.2% to 16.7% based on diagnosis, surgical technique, surgical site, intervertebral level, and instrumentation [7].

Perioperative and intraoperative risk factors for SSI after spine surgery include age >60 years, diabetes, obesity, smoking, hypertension, renal failure, osteoporosis, higher estimated blood loss (EBL), transfusions, longer operating time (OT), longer stay (LOS), cerebrospinal fluid leakage, prior surgical intervention in the region, posterior approach surgery, instrumentation, and American Society of Anaesthesiologists' classification III [8-10].

Surgical site infection has been associated with a variety of adverse outcomes in open spine surgery, including sepsis, long-term antibiotic use, multiple hospitalizations, re-surgery, implant malfunction, pseudoarthrosis, and extended hospitalization [11,12]. This is primarily due to the presence of bacteria that colonize the skin or mucosa. The majority of identified factors related to SSIs have been perioperative in nature, including the source of contamination, the duration of the operation, and the placement of a foreign object [13,14]. Saeedinia et al. and Walsh et al. report that in spine surgical procedures, the most common organism found to cause SSI after the procedure was *Staphylococcus aureus*, suggesting that the contamination originated from the skin [15,16].

Preoperative and postoperative risk factor mitigation, as well as prophylactic preoperative and intraoperative measures, are all strategies for reducing SSI during spinal operations. Preventing SSI with evidence-based prevention strategies during spinal surgery has great potential to reduce mortality, morbidity, and healthcare costs [17]. Wound irrigation tries to minimize the microbial load by removing tissue debris, metabolic waste, and tissue exudate from the surgical area before site closure [18]. Interestingly, although proven to be useful in certain surgical specialties, it is not a generally accepted standard-of-care preventative intervention, and some recommendations do not encourage its use to lower the incidence of SSI [19].

The efficacy of various intraoperative wound irrigation (IOWI) fluids in spinal surgery has not been extensively evaluated in a limited number of clinical trials. Studies have demonstrated that IOWI fluids can help mitigate the development of SSI during open-spine procedures. Therefore, there is no official guidance from any healthcare provider on the specific type of irrigation water to be used. So, the present study was conducted to determine the effectiveness of gentamicin in wound irrigation for the prevention of SSI.

## Materials and methods

Study design and settings

This hospital-based retrospective study was conducted with 40 patients who underwent elective lumbar spine surgery between March 2021 and March 2023 at the Department of Orthopaedics, RL Jalappa Hospital Centre, Kolar, Karnataka, India. Patients were allocated into two groups. Group A received normal saline with gentamicin (NS-G) as the irrigation fluid, and Group B received only normal saline (NS) as the irrigation fluid. Both groups were assessed for the occurrence of SSI.

Ethical clearance

Data collection was performed after obtaining approval from the Institutional Ethics Committee of Sri Devaraj Urs Medical College, Kolar, Karnataka, India (approval no. DMC/KLR/IEC/186/2022-2023).

Selection criteria 

Patients over the age of 18 who underwent lumbar spinal surgeries were included in the study. Patients with pre-existing central nervous system infections (e.g., epidural abscess, discitis, or spine osteomyelitis) and immunocompromised conditions (e.g., uncontrolled diabetes, long-term steroids, intraoperative dural breach, or CSF leak) were excluded from the study.

Sample size and sampling method

A retrospective study of 80 patients by Inojie et al. [20] observed that the SSI rate was 17.5% in patients who had IOWI with normal saline containing gentamicin and 2.5% for dilute povidone-iodine. Considering the occurrence of SSI as a variable with a 95% confidence interval and 80% power, the estimated sample size will be 40. Between March 2021 and March 2023, all consecutive patients had undergone elective lumbar spine surgery, including IOWI, either with saline containing gentamicin or saline alone.

Data collection procedure

Data were collected from the picture archiving and communication system (PACS), which includes socio-demographics, clinical history, and outcome details of patients. The primary endpoint of the study was the incidence of wound infection. Signs of wound infection were identified through the presence of erythema, induration, pain, and the presence of a positive discharge of serum or contaminated fluid. The incidence of dehiscence was also studied. Risk factors for poor wound healing or infection include patient age, sex, BMI, performance level classified according to the American Society of Anesthesiologists, and wound status (by the Centre for Disease Control and Prevention National Nosocomial Infection Surveillance SSI Risk Index) [21].

Data analysis 

After retrieving data from medical records of patients with a minimum of three months of follow-up, the data were entered into an MS Excel sheet (Microsoft Corp., Redmond, WA, USA) and analyzed using SPSS Statistics version 21.0 (IBM Corp., Armonk, NY, USA). The quantitative variable data were presented as the mean and standard deviation. The quantitative information was presented as frequency and percentage distributions. For dichotomous data, the odds ratio (OR) and 95% confidence intervals (CI) were calculated. For continuous data, the mean difference and 95% CI were calculated. The chi-square test was used to assess statistical heterogeneity. A p-value > 0.1 pointed to statistical heterogeneity.

## Results

Of the participants, 60% were males and 40% were females. In both groups, there were more males than females. Overall, the mean age of study participants was 61.53 + 10.49 years. There was no statistically significant difference found between mean age, mean BMI, mean hemoglobin level, mean WBC counts, and mean fasting blood sugar (FBS) levels among both groups. Twelve patients had a history of comorbidities; in Group A, three patients had hypertension, the other three patients had diabetes, and one patient had both comorbidities. In Group B, two patients had hypertension, the other two had diabetes, and only one patient had both comorbidities, as shown in Table 1.

**Table 1:** General characteristics of study participants G+NS: Gentamicin and normal saline, NS: Normal saline, FBS: Fasting blood sugar

Characteristics	Overall prevalence	Group A ( G+NS)	Group B (NS)	T-test	Chi-square test	p-value
Participants	40 (100%)	20 (50%)	20 (50%)	-	-	-
Gender				-	-	-
Male	24 (60%)	13 (65%)	11 (55%)	-	0.416	0.518
Female	16 (40%)	7 (35%)	9 (45%)	-		
Mean age (in years)	61.53 + 10.49	59.7+ 9.9	63.3 + 10.01	1.14	-	0.260
Mean BMI (in kg/m^2^)	24.15 + 2.2	24.12+ 2.07	24.19 + 2.5	0.0964	-	0.923
Comorbidities				-	-	-
Hypertension	5 (12.5%)	3 (15%)	2 (10%)	-	0.543	0.909
Diabetes mellitus	5 (12.5%)	3 (15%)	2 (10%)	-		
Both	2 (5%)	1 (5%)	1 (5%)	-		
None	28 (70%)	13 (65%)	15 (75%)	-		
Mean HB ( gm/dl)	13.4 + 1.9	12.85 + 1.7	13.96 + 1.9	1.947	-	0.058
Mean WBC (cells/mm^3^)	7047 + 2443.1	7001.2 + 2644.2	7093.4 + 2293.3	0.117	-	0.906
Mean FBS (mg/dl)	101.9 + 14.6	102.8 + 13.6	100.4 + 15.8	0.514	-	0.609

**Figure 1:**
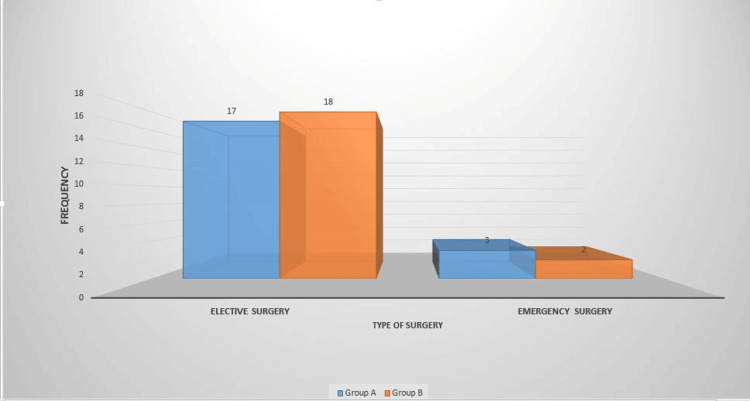
Type of surgery among patients (n = 40)

Among the study participants in Group A, 17 patients had elective surgery, while three patients had emergency surgery. In Group B, 18 patients had elective surgery, while two patients had emergency surgery (Figure 1).

All patients underwent posterior lumbar spine surgery under general anesthesia. A prophylactic antibiotic (1g of cefuroxime) was given during the induction of anesthesia and repeated for surgeries that lasted longer than three hours or for blood loss greater than 1 L. Cleaning and sterile draping techniques were properly followed. All the standard aseptic techniques during surgical procedures were strictly followed in every case.

At the time of wound closure, the surgeon carried out an IOWI using any of the two types of irrigation fluid regimens. Patients in Group A had their spine wound irrigated with NS-G solution (1 L of normal saline mixed with 80 mg of gentamicin injection) in a quantity sufficient to fill the wound and retained in the incision for a minimum of three minutes. After which, the wound was flushed with the remaining NS-G solution. Group B patients had their wounds irrigated with a liter of normal saline alone.

The overall prevalence of SSI among patients was 25%. In total, 17.5% of study participants had superficial SSI, while 7.5% of study participants had deep SSI in the study. A statistically significant difference was found between the mean duration of developing SSI (in days), as shown in Table 2.

**Table 2:** Distribution of variables regarding SSI among patients * Yate's correction applied SSI: Surgical site infection

Variables	Overall prevalence	Group A	Group B	T-test	Chi-square test	p-value
Prevalence of SSI	10 (25%)	3 (15%)	7 (35%)	-	2.133	0.144
Type of SSI	-	-	-	-	-	-
Superficial	7 (17.5%)	2 (10%)	5 (25%)	-	2.15	0.871*
Deep	3 (7.5%)	1 (5%)	2 (10%)	-		
Mean duration of developing SSI (in days)	10.7 + 2.4	9.86 + 2.11	12.67 + 2.08	4.241	-	0.0001

Nine study participants' bacterial cultures showed growth. In Group A, one patient had *Pseudomonas*, and another patient had *Staphylococcus* growth. In Group B, two patients had *Pseudomonas*, five patients had *Staphylococcus* growth, and another one had a negative culture (Table 3).

**Table 3:** Distribution of bacterial culture findings among patients

Bacterial culture	Overall prevalence	Group A	Group B
*Pseudomonas* species	3 (7.5%)	1 (5%)	2 (10%)
*Staphylococcal *species	6 (15%)	1 (5%)	5 (25%)
Negative culture	1 (2.5%)	0 (0%)	1 (5%)

**Figure 2:**
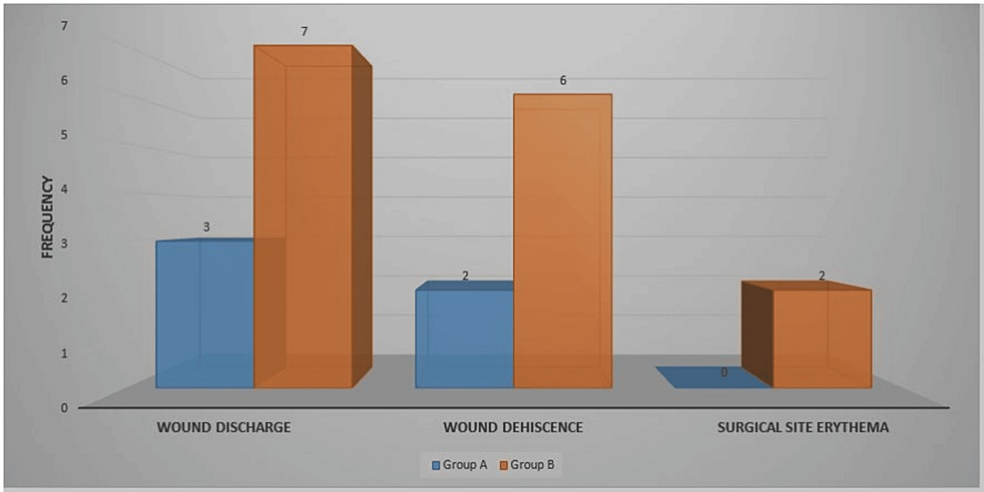
Clinical criteria for SSI (n = 40) SSI: Surgical site infection

In total, 10 patients had wound discharge, eight had wound dehiscence, and two had surgical site erythema. Group B had more signs of SSI than Group A (Figure 2).

## Discussion

In clinical practice, SSI following lumbar spine surgery is a common occurrence. Its incidence has been linked to increased morbidity and death rates, as well as higher healthcare expenses [22,23]. The various risk factors identified are: the patient's age, obesity, diabetes, urinary incontinence, tobacco use or a prior history of smoking, poor nutritional status, complete neurologic deficits, an American Society of Anaesthesiologists score > 2, prior urinary tract infections (UTIs), hypertension, unintended durotomies, operating level, revision surgery, use of non-steroidal anti-inflammatory drugs (NSAIDs), posterior surgical approach, oncologic cases, increased estimated blood loss, blood transfusion, increased operative time, extended sacrum, and spinal instrumentation.

Hospitals in rural and semi-urban areas serve 60% to 70% of the Indian population. However, there is limited information from rural India on SSI prevention strategies and protocols and SSI rates [24-26]. Tertiary hospitals and medical colleges in metropolitan areas of India have been the main focus of national and international collaborative studies on SSIs [27].

In the present study, the overall prevalence of SSI among patients was 25%. In Watanabe et al.'s [28] study, the prevalence of SSI was 6.3%. In a study by Algamdi et al. [29], the prevalence of SSI was 4%. The difference in the prevalence of SSI is due to different admission rates among hospitals and other factors. A study conducted by Bellusse et al. further identified risk factors associated with an increased likelihood of developing an SSI: extended hospitalization, high BMI, transfusion frequency, and the duration of the surgery [30]. To better understand the risk factors associated with SSI and to develop effective prevention strategies, Rao et al. performed a study to determine whether open suction drains increase the risk of SSI following spinal fusion procedures [31].

The study by Inojie et al. [21] featured more males than females, too. In the current study, the mean age of study participants was 61.53 + 10.49 years. Whereas in Inojie et al.'s study, the mean age of study participants was 56.45 years. Similar to our study, no statistically significant difference was found between mean age, mean BMI, mean hemoglobin level, mean WBC counts, and mean FBS levels among both groups.

Normal saline is a commonly used fluid for surgical site irrigation. According to Watanabe et al., the use of a sufficient volume of saline (average > 2000 ml/hour) for irrigation is associated with a statistically significant reduction in SSI (OR = 0.08; 95% CI = 0.01-0.61; p = 0.015) [28]. Another study by Tipper et al. examined saline delivered via pulsed lavage for a total of 3 L [32]. This was coupled with a substantial protocol that was carried out in a single-center trial; thus, it is impossible to make individual statistical inferences regarding the effect of irrigation liquid and technique alone.

Aside from the utilization of the antibiotic in one group and not the other, no other characteristics in the patients' or surgical variables changed substantially between the two treatment groups. As a result, this demonstrated that in lumbar spine surgery, IOWI fluid with an NS-G solution is more effective than only NS. This implies that topical gentamicin solution has greater antibacterial action.

In our study, the bacterial cultures of nine participants showed growth; three had *Pseudomonas* and six had *Staphylococcus*. Previous studies have shown that prolonged surgery, prolonged hospital stays, and poor postoperative wound care are likely to result in SSIs [33]. A study by Hamadeh et al. [34] found that *Staphylococcus aureus* was the most frequent organism found in the bacterial cultures of 22 patients with postoperative posterior spinal infections. Other recurring organisms were *Staphylococcus epidermis*, *Streptococcus*, *Enterobacter cloacae*, and *Bacteroides*. Our Group B patients who had IOWI with the NS solution alone had more signs of SSI than those in Group A. Similar findings were reported in a study by Fang et al. [35].

The findings of this study provide valuable information that may help orthopedic surgeons choose optimal IOWI fluid regimes for SSI prevention. However, we recommend going with a local multicenter one-year follow-up analysis of IOWI in lumbar spinal surgery to evaluate if these advantages are maintained in instrumented spine surgery, where the risk and morbidity of SSI are higher, and this will also allow us to draw more acceptable generalizations. 

Limitations

This is a hospital-based retrospective study, so the chances of bias are higher because of the limited data. Also, no long-term follow-up of patients was done.

## Conclusions

Even though IOWI is a common treatment in everyday surgical practice, the lack of procedural standardization leads to considerable variability, which lowers the quality of the evidence in the available studies. Existing research frequently yields contentious findings. Any changes in the incidence of SSI between different irrigants, particularly those that are antibacterial against those that are not, should be considered with skepticism. Gentamicin is an effective bactericidal, especially against gram-positive organisms such as *Staphylococcus*, the commonest pathogen causing SSI in spine surgery.
